# MAKING THE AGING EXPERIENCE WORTHWHILE: HOW SOCIAL INTERACTION CONTRIBUTES TO ACTIVE AGING

**DOI:** 10.1093/geroni/igz038.1959

**Published:** 2019-11-08

**Authors:** Daniel Doh, Kwadwo Adusei-Asante, Vicki Banham

**Affiliations:** 1 Western Sydney University, Sydney, Australia; 2 Edith Cowan University, Joondalup, Perth, Australia; 3 Edith Cowan University, WA, Joondalup, Perth, Australia

## Abstract

In most parts of the world, people are now living longer lives, which presents both opportunities and concerns over how to make the ageing process a worthwhile experience. The World Health Organisation’s Active Ageing model became a prominent global policy response since 2002 and has evolved into different country-level ageing policies. While a considerable volume of literature exists on active ageing – testing the validity of its various components, there is limited empirical evidence of how social interaction contributes to active ageing for older people and how it can be promoted through policy. In this paper, we examine social interaction and how it contributes to lived experiences of active ageing among a sample of 30 older Ghanaians living in Australia and Ghana. Our findings confirm the significance of social interaction for active ageing, and shows that social interaction creates a sense of purpose for living, which leads to the ability of the individual to build resilience, which mitigates anxieties and pains associated with ill health (especially for frail older people); enhances self-motivation for play and fun; empowers the individual to explore opportunities for continuous activity including leisure, and improves the general feeling of happiness resulting in active ageing – quality of life. The paper’s main argument is that social interaction presents potentials for improving the quality of life (active ageing) for older people and needs to be carefully considered in policy, research and practice.

